# Extended IgE profile based on an allergen macroarray: a novel tool for precision medicine in allergy diagnosis

**DOI:** 10.1186/s40413-018-0186-3

**Published:** 2018-04-26

**Authors:** Enrico Heffler, Francesca Puggioni, Silvia Peveri, Marcello Montagni, Giorgio Walter Canonica, Giovanni Melioli

**Affiliations:** 1 Personalized Medicine, Asthma and Allergy Unit, Humanitas Clinical and Research Center, 20089 Rozzano MI, Italy; 2 Department of Biomedical Sciences, Humanitas University, Via Manzoni 113, 20089 Rozzano MI, Italy; 3 Unità Operativa Speciale Dipartimentale di Allergologia, Ospedale G. da Saliceto, Piacenza, Italy

**Keywords:** Allergen extract, Allergen component, ISAC, ALEX, IgE assay, Laboratory methods

## Abstract

**Background:**

Precision medicine (PM) is changing the scope of allergy diagnosis and treatment. An in vitro IgE assay, a prototype PM method, was developed in the sixties and has garnered increasing interest because of the introduction of recombinant components in the test. More recently, microarrays of allergen components have significantly improved the ability to describe the IgE profile. Aim of this study was to evaluate the characteristics of the newly developed Allergy Explorer (ALEX), a macroarray containing both extracted “whole” allergens and molecular components. This method allows the acquisition of an IgE profile comprising 282 reagents (157 allergen extracts and 125 components), resulting in the widest screening of potential allergens available.

**Methods:**

Sera from 43 patients with allergies were assayed with ALEX and then with ImmunoCAP ISAC. The results of the two tests were compared, and the consistency of the molecular results with the presence of IgE in the relevant extract was also evaluated.

**Results:**

A good correlation between ISAC and ALEX was observed. The ALEX results for second-level tests (i.e., specific IgE to complete extracted allergens) were consistent with the results obtained for the relevant components.

**Discussion:**

Despite differences in the methodology, the IgE profiles detected for molecular allergens by ALEX and ISAC were very similar. The differences were mainly related to the lower dynamic range of ALEX and to the use of a CCD inhibitor in the first incubation phase, which reduced the binding of IgE to CCD, as represented in the extracted allergens and components.

**Conclusion:**

Based on our findings, ALEX is a novel tool for describing the IgE profile in a PM setting, where the IgE assay must be performed on many allergens and components. In particular, polysensitized patients and patients with pollen-food syndrome will have a real advantage due the combination of the second and third levels of allergy diagnostics in the same chip.

## Background

Precision medicine (PM) has a relevant impact on many human sciences and a special impact on the diagnosis and treatment of allergic diseases [1]. Indeed, since its origin, in vivo and in vitro diagnostics have facilitated the accurate and personal treatment of the patient, resulting in a sort of progenitor of PM [2, 3]. Specific IgE analysis was developed in the sixties [4], but in the early nineties, a number of molecular allergens, cloned or obtained by biochemical purification [5], have significantly improved the quality of allergy diagnostics. Indeed, genuine sensitization is identified by the detection of IgE specific for components restricted to a given allergen. A cross-reaction is detected by the presence of an immune response to cross-reacting components, such as profilins and PR-10 [6]. In addition, molecular allergy diagnosis (MAD) allows the detection of IgE specific for “potentially dangerous” components (such as lipid transfer proteins) or apparently safe (or minimally dangerous) components, such as profilins and polcalcins [7]. International guidelines [8] still indicate that clinical history, physical examination and the Skin Prick Test (SPT) are the starting procedures (first level) of every allergy diagnosis (a top-down approach). Specific IgE assay performed on extracted (whole) allergens is considered a second-level diagnostic measure, and MAD is considered a third-level diagnostic [7, 9]. However, other authors suggested that a bottom-up diagnostic approach may also have advantages [10]. In this context, wide IgE profiling based on an allergen microarray (AMA) could be extremely useful. AMA was developed in early 2000 [11], and currently, ImmunoCAP ISAC (Thermo Fisher), based on 112 different molecular components (both extracted and recombinant), is the most studied and most frequently used molecular diagnostic tool based on a microarray [12]. A chip combining second- and third-level diagnostics has recently been developed by MacroArrayDX (Wien, Austria). This chip contains 157 allergen extracts and 125 molecular components and seems to be the widest allergen array currently available. In addition, basic IgE analysis on the allergen extracts is combined in the same test with the evaluation of IgE directed to relevant specific and cross-reactive components. Finally, the inhibition of CCD reactivity further improves the specificity of the IgE assay [13]. In the present study, we describe how this extended IgE profile can be considered a promising tool to support strategies of diagnosis and the treatment of modern PM in in allergic patients.

## Methods

ALEX was developed by MacroArrayDX (Wien, Austria). This array contains 282 reagents (157 extractive allergens and 125 molecular components). The large majority of inhalant, food, latex and Hymenoptera allergen families are represented (Table 1). The test is commercially available, having attained CE certification, which, based on the Council Directive 93/42/EEC concerning medical devices [14], assures that the quality of the assay (i.e., limit of detection, precision and repeatability, absence of possible interferences caused by hemolysis and high levels of triglycerides, absence of an effect of high levels of total IgE, specificity and linearity) is in line with in vitro diagnostic (IVD) features. The different allergens and components are spotted onto a nitrocellulose membrane in a cartridge chip, which is then incubated with 0.5 mL of a 1:5 dilution of serum under agitation. Notably, the serum diluent contains a CCD inhibitor. After incubation for two hours, the chips are extensively washed, and a pretitered dilution of anti-human IgE labeled with alkaline phosphatase is added and incubated for 30 min. Following another cycle of extensive washing, the enzyme substrate is added, and after a few minutes, the reaction is complete. The membranes are dried, and the intensity of the color reaction for each allergen spot is measured by a CCD camera. The dedicated software digitalizes the images and prepares a report that lists the allergens and components and their score in kU_A_/mL. Total IgE is also measured. Finally, an arbitrary calibration curve is obtained by reacting four spots with decreasing concentrations of specific IgE corresponding to < 0.3 kU_A_/L, 0.3 - 1 kU_A_/L, 1 - 5 kU_A_/L, 5 - 15 kU_A_/L and > 15 kU_A_/mL.

**Table 1:** Composition of the allergens available on ALEX

	Total number	Number of extracts	Number of molecular components		Total number	Number of extracts	Number of molecular components
Animals	6	5	2	Fishes	5	3	2
CCD	1	1	2	Foods	23	17	6
Grasses	26	13	13	Fruits	28	21	7
Mites	24	9	15	Legumes	4	4	0
Molds	11	6	10	Meats	0	0	0
Pets	10	3	6	Milks	11	6	5
Trees	25	14	10	Seeds	27	10	17
Weeds	22	15	7	Shellfishes	10	9	1
Eggs	7	2	5	Latex	7	1	6
Extras	21	14	6	Venoms	9	4	5

For the evaluation of the IgE profile in sera from patients with allergies, forty-three serum samples were analyzed by the novel assay. The sample size was calculated considering that, in preliminary assays, 12% of the allergens tested (including low score results) were different when assayed with ALEX and when assayed with other methods (such as specific IgE for extracts or components). Starting from this prestudy evidence, with a confidence level of 95% and a standard error of 0.05, the calculated sample size resulted in 43 different sera. Due to the large amount of allergen families (perennial or seasonal inhalants, food, etc.) and the virtual impossibility of studying all the possible families in a single work, sera with certain characteristics were selected. Therefore, samples from patients with a known reactivity to grasses (where the largest number of molecular components was available) and cross-reacting components, particularly PR-10, profilins and LTPs, were used [15]. In this context, it was considered that the added value of molecular diagnostics could be extensively described. All sera were previously tested with ImmunoCAP ISAC. Since ALEX is a commercially available method, patients (from the private medical practice of one of the authors) were warned that their serum would be tested, without cost, with another method that could define their IgE profile in an exhaustive way. All patients accepted the proposal verbally. The following parameters were evaluated: a) correlation between the results of extracts and the results of relevant components in ALEX; b) correlation between the results of ALEX and the results of ImmunoCAP ISAC; c) correlation between the sum of the scores of ISAC and the sum of the scores of ALEX. The second and the third parameters were assayed for only the components represented in both reagents.

Statistical analysis was performed by using the statistical routines of Microsoft Excel and PAST v3.16, a free software for scientific analysis.

## Results

**Fig. 1:**
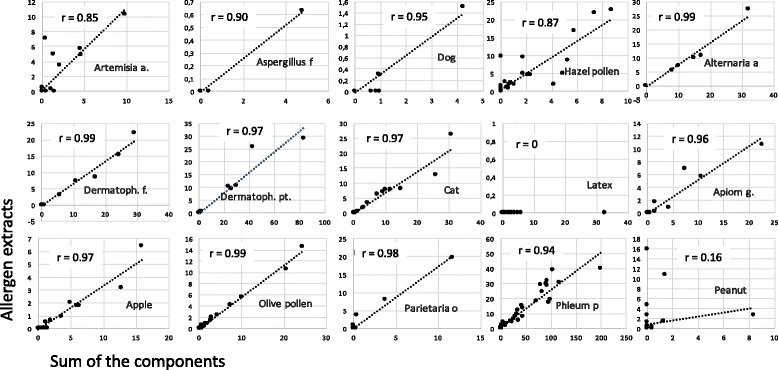
Correlation between the sum of the components (horizontal axis) and the results of the relevant allergen extracts (vertical axis) obtained by ALEX

**Fig. 2:**
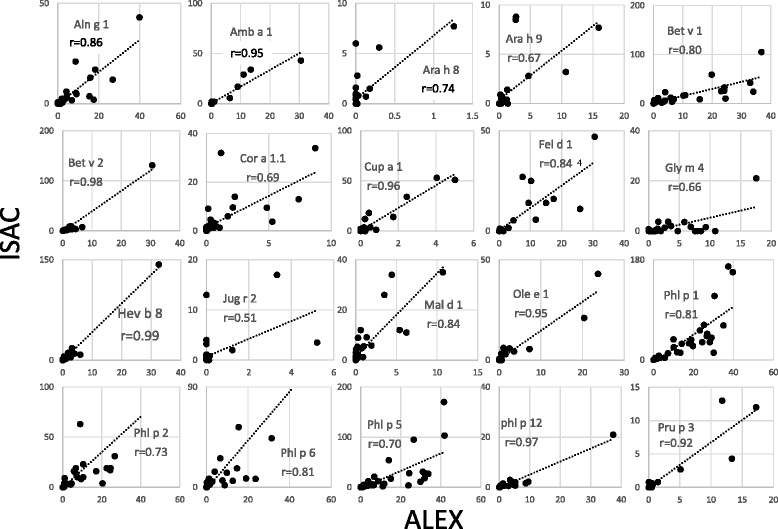
Correlation between the results obtained by ALEX (horizontal axis) and the results obtained by ISAC (vertical axis)

**Fig. 3:**
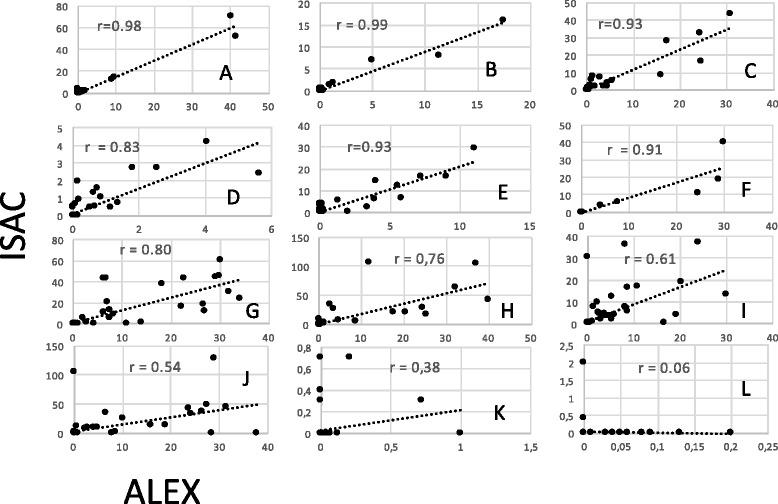
Correlation between the results obtained by ALEX and ISAC at the patient level

**Fig. 4:**
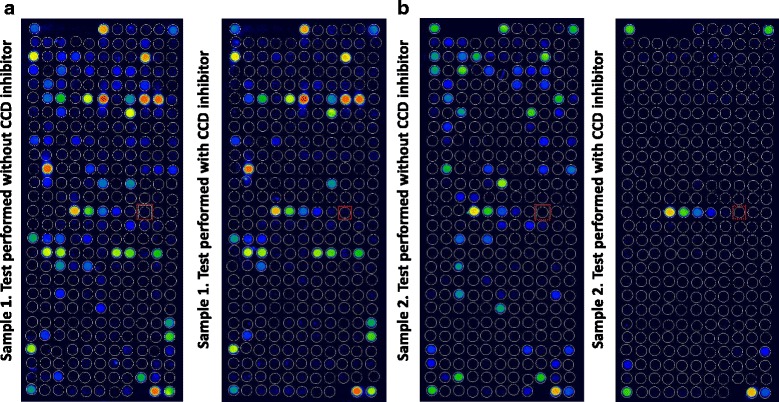
Effects of CCD inhibition on two serum samples assayed by ALEX with or without CCD inhibition. It is evident that after the inhibition of CCD reactivity, the number and the intensity of spots on the chip was strongly reduced in panel **B** (only two spots excluding the calibration curve remained positive). In panel **A**, a less extreme situation was present, where the number and the intensity of spots was reduced but a large number were positive

- Analysis of the consistency of the ALEX results. This analysis was performed using patient sera to identify situations in which the extracts were positive but the component result was negative. A clear consistency was detected for kiwi, alder, ragweed, celery, peanut, mugwort, *Aspergillus fumigatus*, birch, dog, hazel, *Dermatophagoides pteronyssinus, D. farinae*, cat, codfish, hen egg, apple, wall pellitory, timothy grass, peach, and ash (Fig. 1). Poor consistency was observed for Hevea b., where extracts were negative but Hev b 8 (a profilin) was positive in some patients.
- Comparison with the results of ImmunoCAP ISAC. For this aim, two comparisons were made: first, a comparison of the components present in both assays (ISAC and ALEX) and represented in a suitable number in the cohort of patients evaluated, and second, a comparison of the capacity of identifying the same component families. The results of the single-component comparisons are shown (Fig. 2). It is evident that the coefficients of correlation were highly significant for every comparison. Indeed, for this number of comparisons, a value *R* > 0.39 corresponds to a probability of 0.01% for “absence of correlation”, and the lowest value observed was 0.51 for Jug r 2, where the use of the CCD inhibitor in the sample diluent modified the reactivity to a well-known highly glycosylated component [16]. A similar result was achieved by comparing the results by ROC curves (not shown). It is evident that the results closer to the upper left corners indicated that the prediction of both methods was highly comparable.
- Regarding the capacity of ALEX to identify component families, compared to the capacity of ISAC, a statistical analysis was performed, and the results are shown in Table 2. It is evident that certain heterogeneity can be observed, particularly in the frequency of positive results within the analyzed population. For example, the frequency of positive results is higher using ISAC for LTPs, PR-10, profilins and 2S albumins, while ALEX is more frequently positive for tropomyosins, 11S and 7S globulins. Consistently, the dynamic range of ISAC appears, to some extent, to be higher than that of ALEX, at least for certain component families, such as LTPs.
- Another comparison was made by plotting the results of components present in both ALEX and ISAC in the same patient. Figure 3 shows the evaluation of 12 patients representative of the patient cohort. A significant correlation (*r* > 0.39, *p* > 0.01) was observed in 10 out of 12 patients. In a single patient (identified by K), the correlation coefficient R was 0.38 (*p* < 0.02), and in only a single patient (L) could any correlation be observed. However, in these patients, the scores were extremely low and below any clinical or laboratory significance.
- Finally, the effect of the CCD inhibitor was evaluated in some representative samples. Figure 4 shows the ALEX raw data on the macroarray. It is evident that the treatment of sera by the CCD inhibitor results in a sharp decrease of the reactivity to the allergen extracts whose mixture of allergens is characterized by a high concentration of carbohydrate chains in the protein structure. This finding is also true for certain non-recombinant components [13, 17].

**Table 2:** Comparison of percent of positive and mean value in kUA/L for a panel of relevant cross-reacting components assayed by ALEX and by ISAC

	ALEX		ISAC	
Component family	% of positive	Mean value	% of positive	Mean value
LTPs	8.4%	0.43 kUA/L	11.9%	0.71 ISU
PR-10	28.9%	2.06 kUA/L	41.5%	2.84 ISU
Profilins	28.3%	1.46 kUA/L	34.8%	1.43 ISU
Tropomyosins	6.1%	1.44 kUA/L	3.3%	1.11 ISU
11S globulins	2.6%	0.038 kUA/L	0.8%	0.014 ISU
2S albumins	1.0%	0.012 kUA/L	1.5%	1.2 ISU
7S globulins	6.5%	0.16 kUA/L	2.6%	0.30 ISU

## Discussion

In the absence of a gold standard for the evaluation of the performance of an assay of specific IgE, any correlation between different methods should be carefully evaluated. Indeed, the SPT cannot be used to evaluate the results of any serological assay, as it may be positive even in the absence of specific IgE. Specific IgE (assayed on the whole extract) cannot be directly compared with specific IgE measured from the molecular components: indeed, the positivity to the whole extract only rarely corresponds to the positivity of all the relevant components available [12]. Finally, the comparison of assays where recombinant molecules are used may have some pitfalls. Indeed, the clones used to produce the reagents are sometimes different, the folding of these molecules could be different, the immunosorbent used to adhere the component to the solid phase could interfere with the availability of certain epitopes, and finally, specific IgE molecules from different patients show different binding capacities to different epitopes [17]. Moreover, every immunoassay is based on specific concentrations of antigens, test sera, enzyme-labeled antisera and enzyme substrates suitable to offer the best dynamic range under the analytical conditions used. In allergy diagnostics, different platforms and substrates are currently used, and it is normal in laboratory medicine to observe that different serological assays generate different results, even if a correlation is frequently observed under certain operative conditions. In addition, the more sophisticated the assay (or the more complex the antigen or mixture of antigens) is, the greater the heterogeneity of the results.

Having in mind these concepts, in the present study, we analyzed the capacity of ALEX, a novel tool that could be properly used in the bottom-up strategy of allergy diagnostics, to detect sensitization to allergens and components.

To validate ALEX performances, it was considered that this assay was developed on nitrocellulose as an immunosorbent, and the ligation of the allergen to the solid phase was performed by a nanoparticle. Therefore, within the same assay, a comparison of results from the whole extract and the results from relevant allergen-specific components could be accurately performed. A significant correlation between the results of whole extracts with those of the relevant components was observed. This finding is particularly interesting because, in the past, this strict correlation was not completely observed [12]. Some potential explanations for this result include a) the use of the same immunosorbent; b) the choice of representative components; and c) the use of a CCD inhibitor that reduces the non-specific recognition of IgE. The fact that, in a single assay, the allergist can detect positivity to a single extract and obtain information on the relevant components is a real added value.

When molecular components on ALEX were compared with the same components on ISAC, it was considered that the solid phases were different, the serum dilutions were different, the second antibody was probably different, and the enzyme substrate was also different. Additionally, ALEX uses a CCD inhibitor while ISAC does not. Nevertheless, laboratory methods are “artificial” procedures that attempt to mimic in vitro what is suspected to occur in vivo and, more importantly, the results from in vitro tests are used to support the allergist’s diagnosis and therapy. However, despite technical differences, a significant correlation between methods should be achieved. At the component level, the correlation between the results of ALEX and those of ISAC was more than positive, at least for the IgE profiles used in the present preliminary study. Indeed, we focused on samples characterized by a strong IgE reaction against pollens and related cross-reacting allergens because this is an area in which molecular diagnostics seems to offer the most useful results [15]. All the correlations were significant, even if ISAC showed a wider dynamic range. The differences in the dynamic range should be discussed. Indeed, despite decades of using specific IgE in the clinic, the direct correlation between the specific IgE level and the severity of the disease has been observed for certain food allergens in single-plex assays [18]. However, for multi-plexed assays, this correlation has never been described as a rule for all allergens and does not seem to have a proven value in the clinic. On the basis of the observed results, it cannot be concluded that differences in the dynamic range have a significant effect on the performances of the assays.

The capacity to detect sensitization to component families was characterized by a certain heterogeneity. Possible explanations are that at the component level, different molecules were used in the two methods, resulting in a different capacity of sera to recognize different epitopes. In addition, the use of a CCD-inhibitor in ALEX may generate further differences. Finally, the strict correlation between the results of molecular components at a single patient level is the final evidence that ALEX performs similarly to ISAC.

The role of the CCD inhibitor is interesting [13]. Allergists are arguing the role of CCD in human pathology. From an analytical point of view, cross-reactions to CCD are frequent and could impact the decision to start a specific AIT [9, 19, 20]. Thus, the presence of a CCD inhibitor allows a positive result only when the recognition of the allergen (or the component) is specific for the protein itself.

One of the principal added values of ALEX is its capacity to provide results on whole extracts and relevant components within the same assay*.* The combination of second- and third-level assays in the same test allows us to define, in a single hit, the presence of IgE sensitization and whether the reaction is genuine or cross-reactive. Considering the overall social and personal costs, the availability of all the results in a single analytical session has unequivocal advantages. This seems particularly interesting considering that the raw cost of a single allergen or component on the ALEX chip is approximately 0.30 €. Despite the fact that this may be disturbing for some allergists [21], the advantage of having a wide array of allergens and components also allows them to manage the patient using a bottom-up strategy: in this context, 282 allergens in a single chip facilitated an assessment of sensitizations, which was rarely (or never) tested in vitro and/or in vivo in the past. Thus, this feature allows the allergist to better define the IgE profile of the patient, and in certain cases, to improve the identification of the therapeutic strategy, particularly in food allergies. Along this line, it should be considered that the allergen and component selection made by the producers seems to be almost exhaustive. However, if some component, such as omega-5-gliadin, Tri a 14 and alpha-Gal, is inserted in the assay, the diagnostic power of this tool could be further improved.

## Conclusion

In conclusion, ALEX, the immunoassay for specific IgE to whole allergens and relevant molecular components, is an interesting new approach to the bottom-up [10] diagnosis of allergies. The combination of extracts and components should save time and costs when an accurate allergy diagnosis is required, particularly considering AIT for polysensitized patients and patients with pollen/food syndromes. These features, together with the interesting results observed in the present study, show promise that this approach will capture the interest of allergists, particularly molecular allergists, in the near future, because of its direct impact on the management of patients with allergies in the context of a PM approach [1].

## Abbreviations

AIT: Allergen Immunotherapy
ALEX: Allergy Explorer
AMA: Allergen Microarray
CCD: Cross-Reactive Carbohydrate Determinants
ISAC: Immuno-Solid-Phase Allergen Chip
MAD: Molecular Allergy Diagnosis
PM: Precision Medicine
SPT: Skin Prick Test
